# Draft Genome Sequence of the Xanthan Producer *Xanthomonas campestris* LMG 8031

**DOI:** 10.1128/genomeA.01069-16

**Published:** 2016-10-27

**Authors:** Jochen Schmid, Christopher Huptas, Mareike Wenning

**Affiliations:** a Chair of Chemistry of Biogenic Resources, Technical University of Munich, Straubing, Germany; b Chair for Microbial Ecology, Technical University of Munich, Freising, Germany

## Abstract

Here, we report the draft genome sequence of *Xanthomonas campestris* LMG 8031, for which nearly no genetic information is available, despite its good xanthan-producing properties. We performed an Illumina-based sequencing approach of LMG 8031. The genome revealed a 5.0-Mb chromosome having 4,434 coding sequences and a G+C content of 65%.

## GENOME ANNOUNCEMENT

The plant pathogenicity of xanthomonads is mainly based on exopolysaccharide production, which supports adhesion to enable plant penetration, as well as preservation against environmental stress factors such as desiccation or plant protection compounds (1–5). Next to its natural function, xanthan as a highly viscous exopolysaccharide obtained industrial relevance due to its excellent rheological behavior (6, 7). By now, several strains of *Xanthomonas campestris* have been sequenced with a focus on plant pathogenicity: *X. campestris* ATCC 33913 (8), *X. campestris* 8004 (9), *X. campestris* Xca5 (10), *X. campestris* CN14, CN15, and CN16 (11), *X. campestris* 17 (12), as well as *X. campestris* CFBP 1869 and CFBP 5817 (13). Genome sequencing approaches were also performed with a focus on xanthan production: *X. campestris* B100 (14), *X. campestris* JX (15), and *X. campestris* ATCC 13951 (16). To further enhance insights into xanthan biosynthesis of *Xanthomonas* strains with a high production capacity for xanthan, we hereby present the genome of the xanthan-producing strain LMG 8031.

For the determination of the genome sequence of *X. campestris* LMG 8031 we extracted genomic DNA by use of the DNeasy blood and tissue kit (Qiagen, USA) on a culture grown overnight, according to the manufacturer’s protocol (17). Preparation of a library having an average insert size of approximately 750 nucleotides (nt) (IS1) was done as described elsewhere (18). Sequencing was performed on the Illumina MiSeq platform with v3 chemistry. Sequencing data were trimmed and quality-controlled using the NGS QC Toolkit version 2.2.3 (19). High-quality read pairs (2 × 175 nt) were visually inspected using FastQC version 0.11.4 (20) prior to assembly with SPAdes version 2.5.1 (21) applying the *k*-mer combination <21, 33, 55, 77, 99, 127>. The resulting draft genome assembly comprised 50 contigs with an *N*_50_ of 352,468 nt and an assembly size of 5,017,935 nt. Genomic G+C content was 65.09%. With 586,326 high-quality read pairs used for genome assembly, the theoretical sequencing depth is close to 41-fold. Genome annotation was carried out on the RAST server (22, 23), which detected 4,434 coding sequences in 465 subsystems and 55 RNA genes. Additionally, an amount of 20 GGDEF domain–containing proteins were identified manually.

These data will enhance the genomic information of xanthan biosynthesis and facilitate more detailed and comparative analyses of xanthan production and genomic sequences. The xanthan cluster shows an identity of 98% to the biosynthesis operon of strain ATCC 33193 and 99% for *X. campestris* B100, as well as *X. campestris* JX on the nucleotide level.

### Accession number(s).

This whole-genome shotgun project has been deposited at DDBJ/ENA/GenBank under the accession number MBDP00000000. The version described in this paper is the first version, MBDP01000000. Strain LMG 8031 is available from the BCCM/LMG Bacteria Collection (Ghent, Belgium) and from the CIRM–Collection Plant Associated Bacteria/CIRM-CFBP under the accession numbers LMG 8031 and CFBP 1121, respectively.
